# Specialization of Gene Expression during Mouse Brain Development

**DOI:** 10.1371/journal.pcbi.1003185

**Published:** 2013-09-19

**Authors:** Noa Liscovitch, Gal Chechik

**Affiliations:** Gonda Multidisiplinary Brain Research Center, Bar-Ilan University, Ramat Gan, Israel; Indiana University, United States of America

## Abstract

The transcriptome of the brain changes during development, reflecting processes that determine functional specialization of brain regions. We analyzed gene expression, measured using in situ hybridization across the full developing mouse brain, to quantify functional specialization of brain regions. Surprisingly, we found that during the time that the brain becomes anatomically regionalized in early development, transcription specialization actually decreases reaching a low, “neurotypic”, point around birth. This decrease of specialization is brain-wide, and mainly due to biological processes involved in constructing brain circuitry. Regional specialization rises again during post-natal development. This effect is largely due to specialization of plasticity and neural activity processes. Post-natal specialization is particularly significant in the cerebellum, whose expression signature becomes increasingly different from other brain regions. When comparing mouse and human expression patterns, the cerebellar post-natal specialization is also observed in human, but the regionalization of expression in the human Thalamus and Cortex follows a strikingly different profile than in mouse.

## Author Summary

Brain development is one of the most complex biological processes, orchestrated by the precisely timed and coordinated expression of thousands of genes. As the brain develops, specific regions are formed, their structure and function reflected in unique sets of expressed genes. Regional gene expression profiles determine the basic properties of neural systems: controlling how the brain develops from embryos to adults, maintaining the well being of the system, adapting the brain following experience and carrying out specific regional functions. Here we investigate the temporal dynamics of changes in regional gene expression patterns throughout mouse brain development. We identify a *neurotypic* phase around the time of birth, in which patterns of gene expression become more homogeneous across the brain, creating an ‘hourglass’ shaped expression divergence profile. We characterize the biological processes, genes and brain regions responsible for this pattern, and also compare mouse neurodevelopmental expression patterns with parallel data from human, finding striking similarities and differences between the two species.

## Introduction

The development of the nervous system is a highly complex process, involving the coordinated expression of thousands of genes [1]–[3]. Classical models of development describe a process of brain regionalization, that transforms the neural plate through several phases into increasingly refined regions [4], [5]. In the adult, functional compartments of the brain have been shown to exhibit unique transcriptome signatures [6], [7], suggesting that the process of brain regionalization may be accompanied by a similar trend in the transcriptome, where expression profiles become more region-specific as the brain develops.

Regional profiles of gene expression in the brain have been studied extensively. These profiles were used to define new brain delineations based on gene expression [8], conduct comparisons between brains of different species [9], predict neural connectivity [10], [11], capture functional similarities between brain regions [12] and shed light into many aspects of human brain development [2], [3]. Here, we look at changes in regional expression patterns in the mouse brain, aiming to study the specific **timing** of functional specialization. We study expression across 36 developmental neural regions which cover the complete mouse brain at several time points spanning embryonic and post-natal mouse development, and also 41 adult brain regions. Expression was measured for thousands of genes, allowing a large-scale, genomic approach to the study of brain regionalization. We also conduct an inter-species comparison between expression patterns in mouse and human brain development.

Characterizing spatio-temporal patterns of expression can often clarify interactions among genes which seem complex or contradictory, since their measurements are combined across multiple tissues or different ages. This is for example the case with many transcription factors, whose combinatorial cooperation is required for activating transcription of their target genes. Having some factors expressed at a restricted set of brain tissues or regions, can appear as different types of interactions. For instance, transcription factors which are involved in neuronal differentiation, like the bHLH family [13], show both redundant and cooperative interactions [14], [15]. These complex interactions may be explained by different spatial patterns of expression.

This paper studies three aspects of spatio-temporal transcriptome patterns: ***which biological processes*** become spatially specialized, at ***what time*** **points** during development, and in ***which brain regions***. We first trace how expression regionalization changes during brain development. We then identify neural processes that contribute to the regionalization at various developmental phases. Then, we identify the brain regions which become largely dissimilar from other regions, and the genes that contribute to this dissimilarity. Finally, we compare the specialization patterns we find in mouse with corresponding patterns measured in human.

## Results

**Figure 1:**
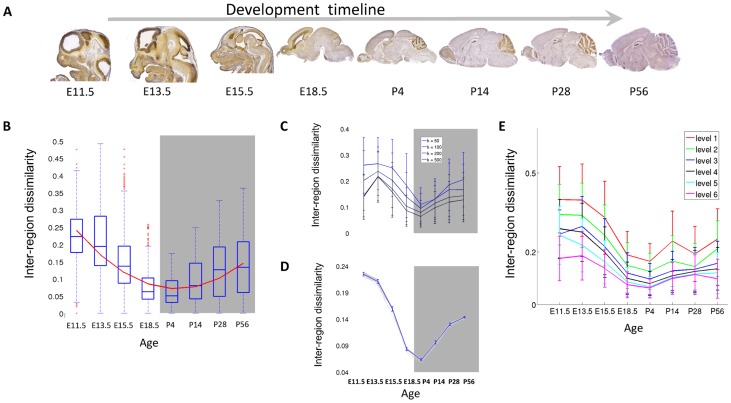
Inter-region distances are minimized around birth. (A) The data: ISH for each gene was performed at eight time points during development. Shown here are mid-sagittal slices for the gene *Hmgn2*, taken with permission from Allen Institute for Brain Science. Allen Mouse Brain Atlas [Internet] Available from: http://mouse.brain-map.org/ [17] (B) Mean pair-wise dissimilarities between the regions. The curve is a second-order polynomial which minimizes the squared error of the fit to the data. Error bars encompass data within 1.5 times the inter-quartile range, and the boxes show the lower and upper quartiles together with the median. (C) The hourglass shape is robust throughout the dataset: Inter-region distance curve was calculated for the data withholding top k most variable genes for each time point. Error bars represent standard error between brain regions. (D) The hourglass shape is robust throughout the brain. Inter-region distance curve was calculated for the data withholding one region at a time. The blue curve is the mean across brain regions, error bars represent standard deviations from mean. (E) The dissimilarity curve using sets of regions taken from different levels of the reference atlas regional ontology tree, starting from the leaf regions (level 1).

To study gene expression specialization during development, we analyze expression primarily based on *in situ hybridization* (ISH) expression values obtained from the Allen Developing Mouse Brain Atlas (devABA) [16]. In this data, mRNA transcript levels were measured for 2002 genes of special interest in brain development at 7 developmental time-points spanning embryonic (E11.5, E13.5, E15.5, E18.5) and post-natal phases (P4, P14, P28). We added another time point, P56, using expression measurements for the same set of genes from the Allen Adult Mouse Brain Atlas [17] (Figure 1A). The genes in the dataset, comprising around 10% of the mouse genome, were selected to include transcription factors, neurotransmitters, neuroanatomical markers, genes important in brain development and genes of general interest in neuroscience (see Methods and supplemental Table S1). We used per-region data that was quantified from ISH images by combining all pixels with the same regional label, based on a mapping of each image to a reference atlas made available by the Allen institute (http://www.brain-map.org). We analyze data from 36 anatomically-delineated regions of the developing brain and 41 regions of the adult brain. These regions encompass the entire brain and are listed in supplemental Table S2 (see methods). The data and pre-processing are described in more details in the Methods section. The data is readily available for download at http://chechiklab.biu.ac.il/~lior/cerebellum.html.

### Changes in expression regionalization during development

Aiming to understand how the transcriptome becomes specialized across different brain regions, we first quantify the differences between expression profiles of brain regions, and examine how these differences change during development.

We quantify the differences between brain regions in terms of the correlation between their gene expression profiles. Specifically, for every pair of regions R_1_, R_2_, we represent each region as a vector of expression levels, calculate their Pearson Correlation Coefficient (PCC) and compute 1- PCC as the dissimilarity between the regions. Figure 1B depicts the mean dissimilarity for each time point across all pairs of brain regions. The dissimilarity varies significantly between ages (*p*-value *<10^−16^*, ANOVA), and its overall profile follows an ‘hourglass’ shape. During early development, the dissimilarity is actually reduced, reaching its lowest value around birth (in E18.5 and P4), although one would expect that the process of region specialization would lead to an increase in dissimilarity in early embryonic development. After birth, the dissimilarity rises again. The variance of inter-region dissimilarity follows the changes in the mean dissimilarity and decreases around birth as well. Interestingly, similar hourglass shapes were also observed in the profiles of transcriptome variability across species during early development, providing striking molecular evidence to the ‘phylotypic stage’ hypothesis [18], [19]. The reduction in expression specialization across brain regions suggests a *neurotypic* phase around birth in which all brain regions tend to have a more similar transcriptome.

To test if the overall hourglass shape is a wide effect or strongly depends on a small set of genes, we also measured the dissimilarity using 100 random subsets of sizes K = 1000, 500, 200 and 100 genes. We find that the hourglass shape is largely insensitive to the subset of genes analyzed (supplemental Figure S1). To further ensure that the hourglass effect is not driven by a small number of highly variable genes, we measured again the dissimilarity, this time after removing the genes with the largest inter-region variability for each time point. At each time point, we measured the standard deviation across regions for every gene, and removed the top *k* genes with the highest standard deviation values (k = 50, 100, 200, 500). The hourglass shape was robust even when removing the 500 most variable genes (25% of the dataset, Figure 1C). We also tested the sensitivity of the hourglass shape to the selection of regions by computing the dissimilarity repeatedly, each time with one region being excluded from the analysis (“leave one region out”, Figure 1D). To test how the delineation of the brain into regions may affect the results, we used the hierarchical structure of the anatomical regions to select six sets of regions at increasing sizes (see Methods). Figure 1E depicts the dissimilarity profiles for each of the six sets, as computed at various resolutions, from 488 developing and 631 adult small brain regions at the most refined level, to 48 developing and 13 adult brain regions at the most coarse level. The hourglass shape of dissimilarity profile is largely preserved in all delineations. Together, these results demonstrate that the hourglass shape is robust throughout the dataset and is not constrained to specific genes or brain regions.

### Functional characteristics of early and post-natal regionalization

Which biological processes could underlie the pattern of inter-region dissimilarity? In principle, the hourglass shape could stem from functions or genes whose individual expression profiles follow the hourglass shape. Alternatively, the shape could be the result of a mix of several biological processes, some contributing to the decreasing phase of the hourglass and some contributing to the increasing phase. To test these alternatives, we created a temporal profile for each gene that quantifies its contribution to the hourglass shape at developmental time points (E11.5 - P28) (see Methods). We then used the *k*-Means clustering algorithm [20] to group the profiles into distinct clusters of genes that have congruent developmental dissimilarity patterns, and searched for functional enrichment in these clusters using Gene Ontology (GO) categories (see Methods).

**Figure 2:**
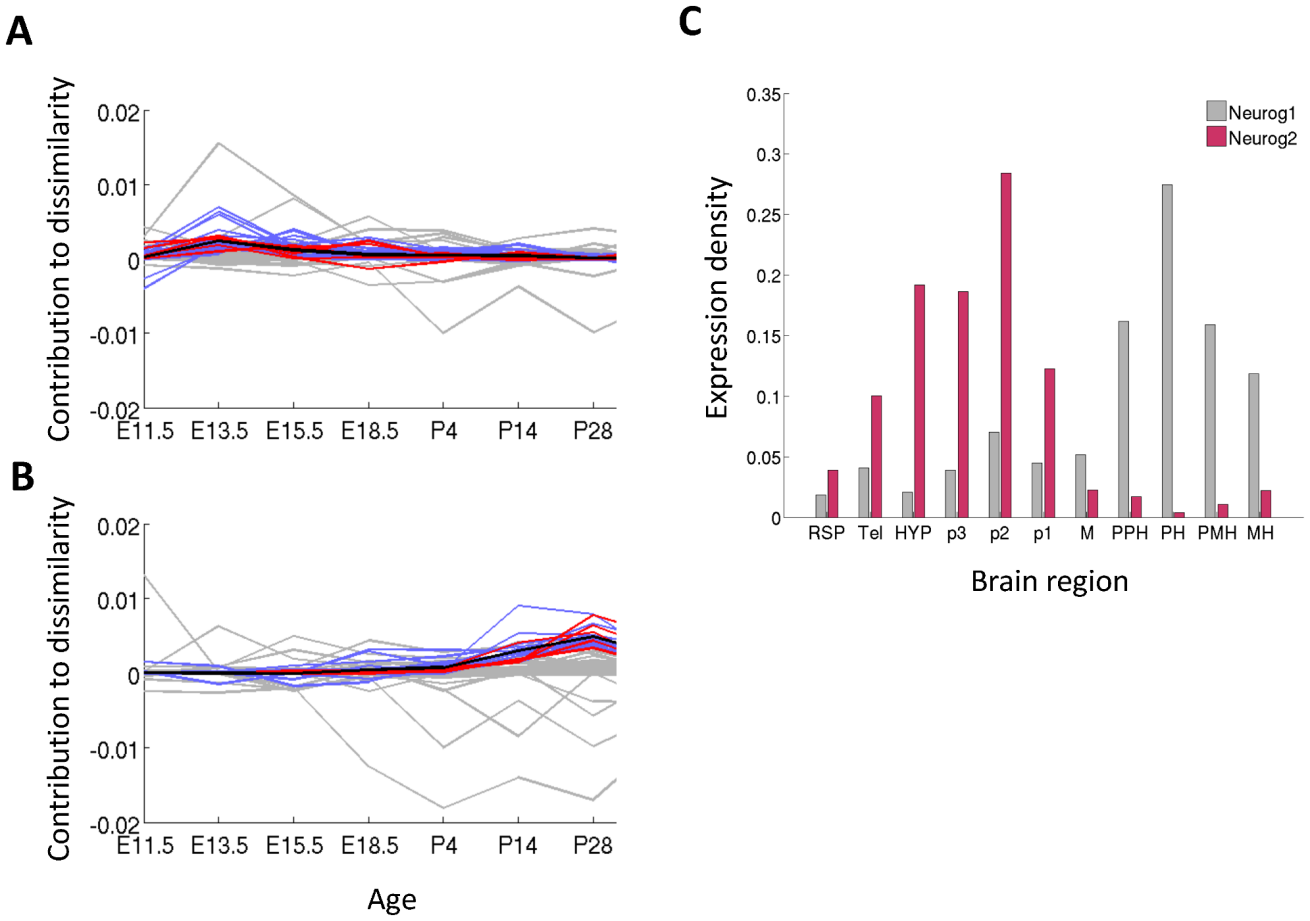
Functional characterization of hourglass shape. (A), (B) Clusters of gene profiles that are functionally enriched. Each profile is a measure of contribution to dissimilarity D (see Methods). Black bold curve is the mean of the cluster. Blue lines - all the genes in the cluster; red lines - genes that are in the cluster and in the category; grey lines - genes that are not in the cluster even though belong to the category. (A) *Neuron migration* shows decreasing dissimilarity (B) *Learning or memory* shows a post-natal increase in dissimilarity. (C) Spatial expression of the genes *Neurog1* and *Neurog2* at E11.5 in 11 coarse regions, selected as *neuron differentiation* genes with highly similar sequence.

**Figure 3:**
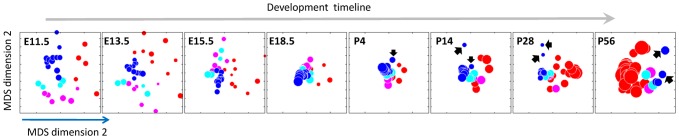
Changes in dissimilarity across individual brain regions. Embedding of all regions onto a 2D plane using multidimensional scaling. Each circle corresponds to a brain region, with a size that corresponds to the within-region expression standard deviation and a color that corresponds to its embryonic origins. Red: forebrain, telencephalon; pink: forebrain, diencephalon; cyan: midbrain; blue: hindbrain. Rhombomere 1 and Isthmus in the developing post-natal time points are and the cerebellar cortex and cerebellar nuclei at P56 are marked with a black arrow.

We found two main families of clusters that were functionally enriched (pFDR, *q*-value <0.01), each family accounting for a different phase of the hourglass shape, and depicted in Figure 2. Genes from the first family contributed largely to the dissimilarity during early embryonic development and are related to nervous-system development categories, such as *neuron differentiation*, *axonogenesis* and *forebrain development* (an example is shown in Figure 2A). At the same time, genes from the second family have a high contribution to dissimilarity in late post-natal developmental time points (P14 and P28) and tend to be related to experience dependent plasticity, with enriched categories such as *regulation of synaptic transmission*, *behavior*, *learning and memory* (Figure 3B). The full list of enriched categories is available at supplemental Table S4.

To quantify the relative contribution of GO categories to early embryonic and late postnatal dissimilarity, we computed a category contribution index (see Methods). The top contributing categories at E11.5 are related to nervous system construction, including *positive regulation of neuroblast proliferation* and *axonogenesis* (top categories are given in Table 1, see the full list in supplemental Table S1). The top scoring categories at P28 are related to the utilization of the nervous system, including *regulation of neurotransmitter secretion* and *visual perception*. An exception to this rule is the category *hindbrain development*, ranked at #10 at P28, which is in agreement with the postnatal timeline of hindbrain development [21].

**Table 1:** Mean contribution values of GO categories at E11.5 and P28.

GO category	contribution at E11.5	GO category	contribution at P28
positive regulation of neuroblast proliferation	0.0022	neurotransmitter metabolic process	0.0011
retinal ganglion cell axon guidance	0.002	regulation of neurotransmitter secretion	0.00039
CNS projection neuron axonogenesis	0.0018	sensory perception of sound	0.00032
central nervous system neuron development	0.0016	regulation of neurotransmitter levels	0.0003
midbrain development	0.0015	sensory percept. of mechanical stimulus	0.00028
central nervous system neuron axonogenesis	0.0015	synaptic transmission, dopaminergic	0.00027
hindbrain development	0.0012	visual perception	0.00027
neural tube development	0.0011	regulation of long-term synaptic plasticity	0.00027
motor axon guidance	0.0011	sensory perception of light stimulus	0.00024
negative regulation of glial cell differentiation	0.0011	hindbrain development	0.00024

The contribution of each GO categories C to inter-region dissimilarity was computed as the mean contribution of all genes assigned to C (see Methods).

The observed expression dissimilarity means that each of these neural processes contains a mixture of genes with different spatial expression patterns. Such spatial differences could result from specialization at the level of gene families: the same process may be carried out in different brain regions using different members of a common gene family. This is for example the case with homeobox genes, well known to operate as pattern specificators in the brain [22], [23].

To search for spatial specialization within gene families of interest, we collected pairs of genes from the 17 enriched GO categories discussed above. We computed both their spatial correlation at developmental ages with peak dissimilarity (E11.5 and P28), and their sequence similarity (see Methods, results summarized in supplemental Table S5). Results for an example category ‘*neuron fate commitment*’ are presented in supplemental Figure S2.

The spatial specialization of genes that are members of the same family, could explain apparent inconsistencies in the way they cooperate, by considering their different spatial patterns.

One interesting example is the pair of paralogs *Neurog1* and *Neurog2*, where there are mixed reports suggesting that they sometimes operate in a synergistic way [14] and sometimes in a redundant way [15]. These genes are bHLH transcription factors involved in neuronal differentiation determination and subtype specification during embryogenesis [13]. Figure 3C shows that they display a complementary pattern of expression at E11.5 (ρ = −0.59, Pearson correlation): Neurog2 is prominently expressed in areas derived from the forebrain, and Neurog1 is expressed more strongly at hindbrain areas. Their different spatial distribution could explain why they were found to be redundant in some conditions, for example, in tissues where both are expressed, but not in all of them.

### Expression conservation across regions and their embryonic origins

To further understand how the changes in dissimilarity relate to the process of regionalization throughout development, we next look into the question of which brain regions contribute to the overall dissimilarity. Brain regions develop from three embryonic vesicles; the prosencephalon (forebrain), mesencephalon (midbrain) and rhombencephalon (hindbrain). In the adult brain, Zapala *et al.* showed that brain regions sharing an embryonic precursor also tend to share similar expression profiles [24]. Here we further examine the dynamic of this relation, testing how the embryonic origins of brain regions influence the changes in their dissimilarity.

Specifically, we first visualize the changes in region dissimilarity over time. All regions were embedded in a two dimensional space, while preserving the pair-wise dissimilarity of their expression profiles (using non-metric multidimensional scaling [20], see Methods). The embeddings for each time point are shown in Figure 3, revealing how the hourglass shape manifests itself across individual regions. In accordance with the hourglass shape, brain regions tend to be less dispersed in the two time points that surround birth (Figure 3, E18.5, P4). To visualize the relation between expression profiles and the embryonic origin of each region, we colored the regions in Figure 3 by their embryonic vesicle of origin. Indeed, regions sharing the same origin tend to be clustered together throughout development. This relation was also statistically significant (ρ = 0.33, *p*<0.05, mean over all time points of Pearson correlation between the dissimilarity and embryonic tree distance).

The regions that are most diverged in the developing post-natal time points are Isthmus and rhombomere 1, the two regions that give rise to the cerebellum (Figure 3, black arrows). In the adult time point, the cerebellar cortex is, notably, the most unique region in the brain in terms of gene expression. These results are to a large extent consistent with previous analysis of cerebellar gene expression [17], [24]. The post natal shift in cerebellar gene expression is also in agreement with the functional role of the cerebellum, since the cerebellum is a motor coordination center that relies on sensory input becoming available only after birth. Cerebellar development is also known to take place at a large part after birth [25].

We next turned to identify the specific genes contributing to the post-natal shift in cerebellar gene expression. We defined the contribution of each gene *g* to the cerebellar dissimilarity, as the difference between the total cerebellar dissimilarity with and without *g* (see Methods), and listed the top twenty genes that contribute most to cerebellar distance at each of the three post-natal developmental time points (supplemental Table S6). Overall, 78% (32/41 unique genes) of the top contributing genes are known to be related to the cerebellum, including genes that play an important role in cerebellar development or function like *Neurod1*, *Pvalb*, *Zic1* and *Zic5*. The remaining top genes (8/41) have not been previously linked to the cerebellum, even though some of them ranked very high in our contribution lists. For instance, heterogeneous nuclear ribonucleoprotein A/B (*Hnrpab*), which is ranked 8 at P4, and microfibrillar-associated protein 4 (*Mfap4*) which is ranked 20 at P4 and 13 at P14. *Hnrpab* is a DNA and RNA binding protein, and is suggested to be involved in cytostatic activity [26]. *Mfap4* is thought to be an extracellular matrix protein which is involved in cell adhesion or intercellular interactions, and has almost no other associated information. Both of these genes make interesting targets for further investigation as important to cerebellar specialization.

### Comparison with human development

The above findings show how the specificity of the regional expression profiles in the brain changes during development. How do these findings generalize to other mammals? A recent study provides a good opportunity to test these findings in humans [3]. Kang and colleagues measured the transcriptome of 57 human subjects using DNA microarrays of 11 cortical regions, the mediodorsal nucleus of the thalamus, striatum, amygdala, hippocampus and the cerebellar cortex.

**Figure 4:**
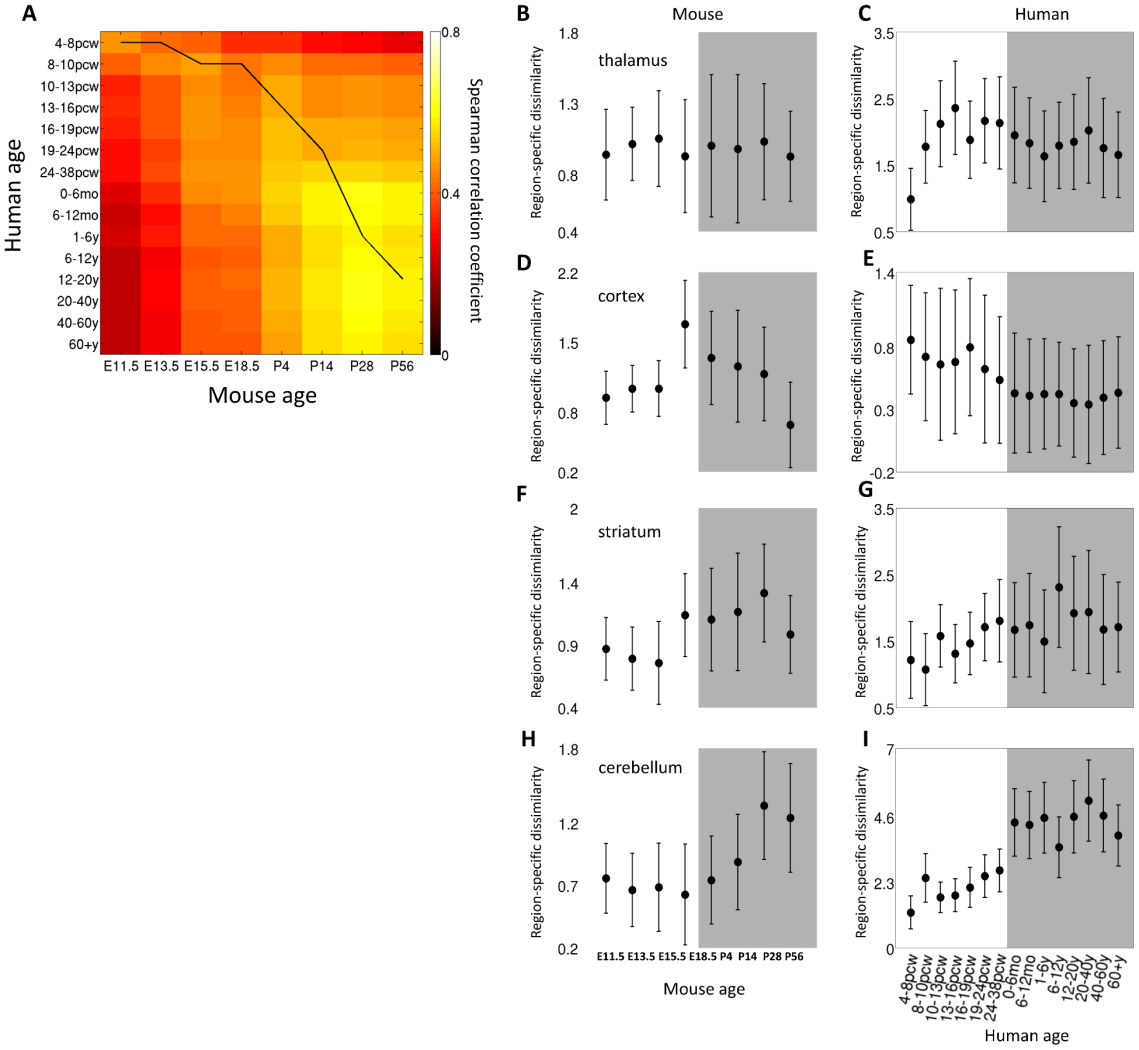
Comparison with human data. (A) Cross correlation between mouse and human gene expression. The black line is taken from known developmental timeline of the two species based on anchor events [27]. Region-specific dissimilarity curves of four brain regions in mouse and human. (B) mouse thalamus, (C) human mediodorsal nucleus of the thalamus, (D) mouse dorsal pallium, (E) human cortical regions, (F) mouse striatum, (G) human striatum, (H) mouse rhombomere 1 and isthmus and (I) human cerebellar cortex. Error bars denote standard deviation across regions.

We first aimed to assess if the gene expression levels in mouse and human can be compared. We considered the human genes that are orthologous to the 2002 mouse genes and computed the Spearman correlation of the gene expression profiles of every pair of time points, averaged over brain regions (see Methods). Figure 4A depicts the cross correlation between the human and the mouse cerebellar developmental timeline, showing a high correlation between the expression profiles of the two species, which peaks along the translation between the mouse and human brain development timelines proposed in [27].

We next turned to compare expression in specific regions of the mouse and human brains, focusing on four mouse brain regions which have parallel regions in the human data (see Methods). The human cortical areas were averaged and compared to the mouse dorsal pallium, the human mediodorsal nucleus of the thalamus was compared to the mouse thalamus, the human cerebellar cortex was compared to two mouse regions which were averaged: rhombomere1 and isthmus, and the human and mouse striatum were compared as well. For each pair of parallel regions, we first looked at the overall temporal correspondence of the mouse and human development timelines by computing the correlation between expression levels of the two species during development. We computed the cross-species correlation as described above for the four pairs of human regions and their parallel mouse regions, finding high correlation values for all region pairs (supplemental Figure S3).

We next looked at region-specific dissimilarity and traced how the dissimilarity of each of the four regions from all other brain regions changes over development, in both mouse and human (see Methods). The specialization patterns in mouse and human show partial correspondence (Figure 4). While the thalamus is specialized very early in human (Figure 4C), at 4–8pcw, in the mouse it keeps a relatively constant distance from the rest of the regions (Figure 4B). In mouse, the cortex is specialized right before birth (Figure 4D), while in human there is a decrease in specialization over time (Figure 4E). The Striatum in mouse gets specialized right before birth (Figure 4F), and in human it keeps a more or less constant distance (Figure 4G). The region with the highest correspondence between mouse and human is the cerebellum, which becomes specialized right after birth in both species (Figure 4H,I). The differences between mouse and human regional specialization is striking, and the fact that the most similar profile is for the cerebellum is especially interesting given the fact the cerebellum shows the lowest inter-species correlations for the post-natal time points (supplemental Figure S3D).

## Discussion

We characterized how the dissimilarity between transcription profiles of brain regions changes during development of the mouse brain. Based on the process of brain regionalization we expected to observe a monotonous increase in transcription specialization, but we actually found that brain regions exhibit increasingly more similar expression profiles during early embryonic development, until reaching a “neurotypical” phase around the time of birth. After birth, brain regions tend to specialize and their expression dissimilarity increases. Functional characterization of the hourglass shape suggests that it is derived from two separate, complementary processes: the embryonic reduction in dissimilarity is dominated by genes responsible for constructing and shaping the brain, while the post-natal increase in specialization largely involves processes that govern the operation of the nervous system, like neural activity and plasticity.

When visualizing the dissimilarity between the regions (Figure 3), it is apparent that the cerebellum “breaks off” from the rest of the regions after birth. The dissimilarity between the cerebellum and other regions grows at each post-natal time point and so does its dissimilarity from other regions of the hindbrain. This dynamic is consistent with the view that cerebellar development follows unique cues from the junction of the midbrain and hindbrain [28], [29], and therefore its transcriptome may differ from other hindbrain regions significantly [30]. Indeed, the cerebellum has been shown before to be the most unique region in terms of its expression profiles [3], [17], [24], [25]. One explanation for the late specialization lies in the main function of the cerebellum as a motor coordination and sensory-motor integration center.

The above findings are in partial accordance to a recent large scale developmental-brain transcriptome study in humans [3], where similarity between brain regions was aggregated across three long life periods: embryonic, postnatal and adult. In both mouse and human dissimilarity decreases before birth. However, on average, the similarity in these periods seems to grow from post-natal development to adulthood in human. In the mouse dataset we see the opposite effect: a robust increase in dissimilarity during post-natal development, following birth. While the cerebellum specializes after birth in both species, other temporal dissimilarity profiles differ between the species. Further measurements are needed to clarify if this mismatch reflects a fundamental difference between rodent and primate development, or if it is due to differences in the experimental technique or the specific subset of six regions measured in humans.

Interestingly, recent studies have shown examples of whole-organism developmental gene expression profiles that follow an hourglass shape. Kalinka et al. measured inter-species distances over development for six species of flies and found that the distance is minimized during the presumed ‘phylotypic’ stage [31]. Domazet-Lošo et al. analyzed the phylotypic stage further by looking into the relative ages of genes expressed in different stages of development and finding that the genes expressed during the phylotypic stage are more ancient, hence more stable in face of evolutionary changes [19].

The above findings suggest that expression dissimilarity decreases at the same developmental phases where brain regions become anatomically segregated and specialized. The question remains if the reduced dissimilarity in mRNA is accompanied by reduced dissimilarity in regional protein abundance profiles across the brain. Alternatively, post-transcription regulation mechanisms may take a larger role in preserving specialization across brain regions and explain this apparent mismatch.

ISH provides a much higher spatial resolution than the one used in this study, that can be used to investigate specialization at a finer scale of cell layers and even cell types. This is especially important when considering the fact that gene expression as measured here reflects cell densities, as well as transcript abundance. Quantifying and correcting for regional cell densities is a crucial step towards a more accurate description of the neural transcriptome. Furthermore, the recent availability of transcription measures from other species [32], [33] calls for a thorough study of the similarities and differences of development as reflected in gene expression between species to understand the genetic blueprint underlying brain development.

## Methods

### Data acquisition and pre-processing

The detailed process of data acquisition was described in [17]. 2002 genes were chosen from five classes: (1) Transcription factors, including homeobox, basic helix-loop-helix, forkhead, nuclear receptor, high mobility group and POU domain genes. (2) Neuropeptides, neurotransmitters, and their receptors. In particular genes involved in dopaminergic, serotonergic, glutamatergic and gabaergic signaling. (3) Neuroanatomical marker genes. (4) Genes relevant to brain development including axon guidance, receptor tyrosine kinases and their ligands. (5) Genes of general interest including common drug targets, ion channels, cell adhesion, genes involved in neurotransmission, G-protein-coupled receptors and genes involved in neurodevelopmental diseases. One animal used to measure expression for each gene.

Brain regions may change dramatically in size and shape causing a problem to compare gene expression in the brain across different developmental stages. Here, expression density for each brain region in each time point was measured while taking the differences in size into account. The expression density for each brain region *R* is defined as the sum of expressing pixels in *R* divided by the total number of pixels that intersect *R* (taken from: http://developingmouse.brain-map.org/docs/InformaticsDataProcessing.pdf). Since expression measurement for each gene come from different individual brains and their 3D shapes differ, this registration process is prone to mistakes, especially for small regions. To avoid errors that stem from erroneous registration, we selected a set of regions that are large relative to the magnitude of the registration perturbation.

### Selecting brain region delineation

We used the hierarchical structure of the anatomical regions as defined in the reference atlas ontologies available in the Allen Brain Atlas website to define six delineations of the brain into sets of regions. These delineations are achieved by considering several levels of the tree in a serial manner. We started with the set of leaf regions and then repeatedly took their “parent” regions five times, yielding six sets of regions corresponding to six levels of the ontology tree. The most refined level has 488 developing and 631 adult small brain regions, and the most coarse level 48 developing and 13 adult brain regions. For some time points, expression measurement are only available for a small number of regions, and the remaining regions were ignored.

### Contribution of individual genes to the hourglass shape and functional analysis

To functionally characterize the hourglass shape, we calculated the contribution of each gene to the inter-region distance as: 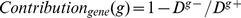 where 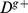 is the mean dissimilarity (1-PCC) across all N pairs of regions
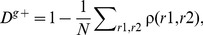
(1)and 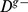 is similarly measured, but after excluding the gene *g*. This was used to create a temporal contribution profile for each gene.

To find biological processes who share similar contribution profiles, we clustered the profiles using k-Means (k = 10, 15, 20, 25, 30, 35, 40, 45, 50). The resulting clusters were tested for Gene Ontology functional enrichment [34]. We limited the analysis to GO categories with at least 10 associated genes in our dataset (∼0.5% of the dataset) and to GO categories related to nervous system structure and function. This was done by taking several top-level categories like *neurological system process* (GO:0050877) and *nervous system development* (GO:0007399) and get all of their descendant categories in the GO graph. We added to this several more biological process categories and cellular component categories with their descendants such as neuron projection, neuronal cell body, synaptosome etc. The full list of categories we used is available as supplemental Table S2.

We tested for enrichment using a hyper-geometric test. P-values were corrected for multiple comparisons using a double-FDR approach: First, for each clustering result, we corrected the enrichment *p*-values using False Discovery Rate (FDR, [35]). Next, to correct for the fact that the clustering was computed for ten different values of k, we corrected the 10 *p*-values each category received using FDR as well. Finally, to present the most refined categories, we screened the resulting categories using the hierarchical structure of the GO tree, and discarded categories that had a descendant category with a lower *p*-value.

To decide if a cluster represents the embryonic or the post-natal dissimilarity (or neither), we pooled all contribution values of genes in the cluster in the embryonic time-points (E11.5, E13.5, E15.5, E18.5) and separately pooled the ones in the post-natal developmental time points (P4, P14, P28). We then applied a Wilcoxon signed-rank test to decide if there is a significant difference between the two samples. If there was, we checked the direction of the difference by comparing the medians of the samples. The results appear in supplemental Table S3, column 3.

The contribution of each GO categories *C* to inter-region dissimilarity was computed as the mean contribution of all genes assigned to *C*. 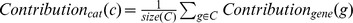. This index captures both large categories and small categories with highly contributing genes.

### Identifying genes with similar sequences

To identify genes from the same gene family we computed the similarity of their protein coding sequence as measured by the Needleman-Wunsch algorithm [36]. We used BLOck SUbstitution Matrix 50 (BLOSUM50) as the scoring matrix for the global alignment and gap alignment penalty of 8. Pairs with a score of zero or higher were considered as matches.

### Visualizing inter-region distances

To visualize the temporal dynamics of the inter-region dissimilarity in the brain, we embedded the regions in a two-dimensional space while preserving the pair-wise dissimilarity of their expression profiles, using non-metric multidimensional scaling. For easier comparison of the time points, at each time point, the location of the regions was adjusted to best match the location of the other regions using MATLAB's ‘procrustes’.

### Dissimilarity of one region to the rest of the brain

To quantify the time course of expression specialization, we measured the dissimilarity between a region *R* and the remaining brain regions. The region-specific index for a region *R* is defined as the average dissimilarity between *R* and all the other regions, 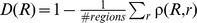, divided by the mean inter-region dissimilarity of Eq (1).

### Mouse-human comparison

To compare expression in mouse and human brains, we focused on four mouse brain regions which have parallel regions in the human data of [3]. Since the mouse cortical regions have data only for P28, we used their parent region, the dorsal pallium, to compare with the 11 human cortical areas, averaged to create one cortical expression profile. The human mediodorsal nucleus of the thalamus was compared to the mouse thalamus, the human cerebellar cortex was compared to two mouse regions which were averaged: rhombomere1 and isthmus, and the human and mouse striatum were compared as well.

To identify human genes that are orthologous to the 2002 genes in the mouse dataset we used the R/BioConductor package BioMart [37]. The full list of the orthologous pairs is available as supplemental Table S5.

The set of 1737 ortholog gene pairs was used to calculate the Spearman correlation between mouse and human expression profiles, averaged over regions, for every two time points (Figure 4A), and also for the four parallel regions (supplemental Figure S3).

## Supporting Information

Figure S1 Robustness of hourglass shape to the selection genes. The dissimilarity curve was computed using random subsets of genes sized (A) 1000, (B) 500, (C) 200 and (D) 100. The shape is preserved and largely remains even when using 100 genes, 5% of the full dataset.
(TIF)

Figure S2 Sequence similarity vs. spatial correlation of gene pairs belonging to the GO category ‘*neuron fate commitment*’. Pairs of genes with sequence similarity >0 and spatial correlation >0.2 are marked in yellow. Pairs of genes with sequence similarity >0 and spatial correlation <−0.2 are marked in red.
(TIF)

Figure S3 Cross-correlation between mouse and human expression profiles over development. Coherence between expression profiles for orthologous genes was measured using Spearman correlation, for every pair of time points in mouse and human. (A) Thalamus (B) Cortex (C) Striatum and (D) Cerebellum. The black line depicts the mapping between neurodevelopmental timelines of the two species proposed by [27].
(TIF)

Table S1 **Gene symbols and entrez-ids of all the genes in the dataset.**
(XLSX)

Table S2 **The neural regions used for the developing brain and the adult brain.**
(XLSX)

Table S3 **Neural-related parent GO categories.** GO categories that were used to screen the GO hierarchy for categories related to nervous system development, structure and function. We used these categories and all of their descendants in the GO tree.
(XLSX)

Table S4 **Functional enrichment of single gene dissimilarity profiles.** List of GO categories that were found to be enriched in clusters showing embryonic or post natal high dissimilarity.
(XLSX)

Table S5 **Sequence similarity and spatial correlation of pairs of genes belonging to 17 GO categories contributing to the ‘hourglass shape’.** These were measured at the two peak dissimilarity time points: E11.5 and P28.
(XLS)

Table S6 **List of mouse and human orthologous pairs used for interspecies comparison.**
(XLSX)

Table S7 **Genes with a high contribution to cerebellar specialization.** List of genes that are in the top 20 contribution values for the developmental post-natal time points (P4, P14, P28). The genes are sorted by their total contribution at these time points. The number of time points where the genes appeared in the top-20 list is also included.
(XLSX)
