# Supplementary figures and images for: Specialization of Gene Expression during Mouse Brain Development
Source: PLoS Comput Biol. 2013 Sep 19;9(9):e1003185. doi: 10.1371/journal.pcbi.1003185 (PMC3777910; doi:10.1371/journal.pcbi.1003185)

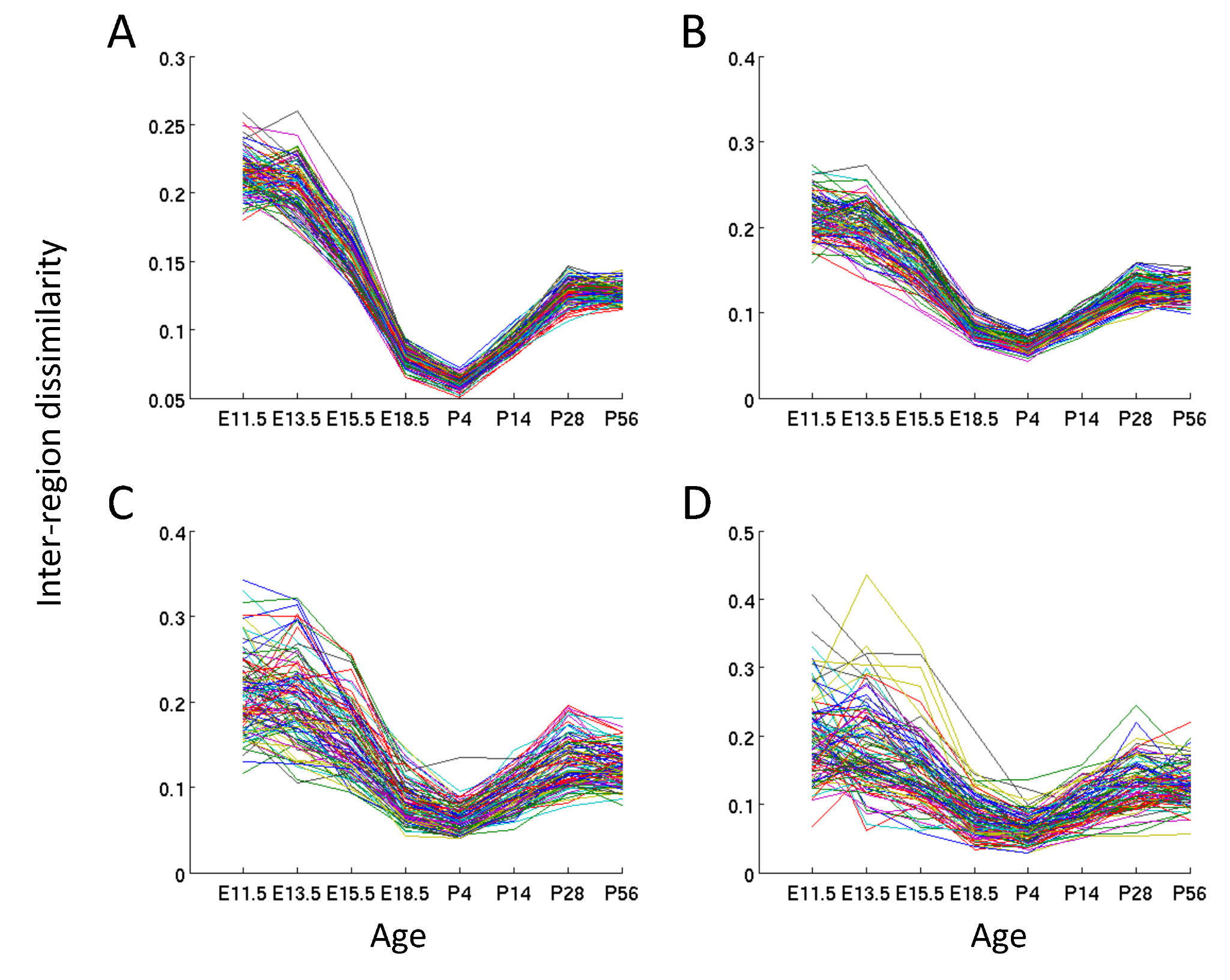

Supplement: Figure S1 — Robustness of hourglass shape to the selection genes. The dissimilarity curve was computed using random subsets of genes sized (A) 1000, (B) 500, (C) 200 and (D) 100. The shape is preserved and largely remains even when using 100 genes, 5% of the full dataset. (TIF) [file pcbi.1003185.s001.tif]

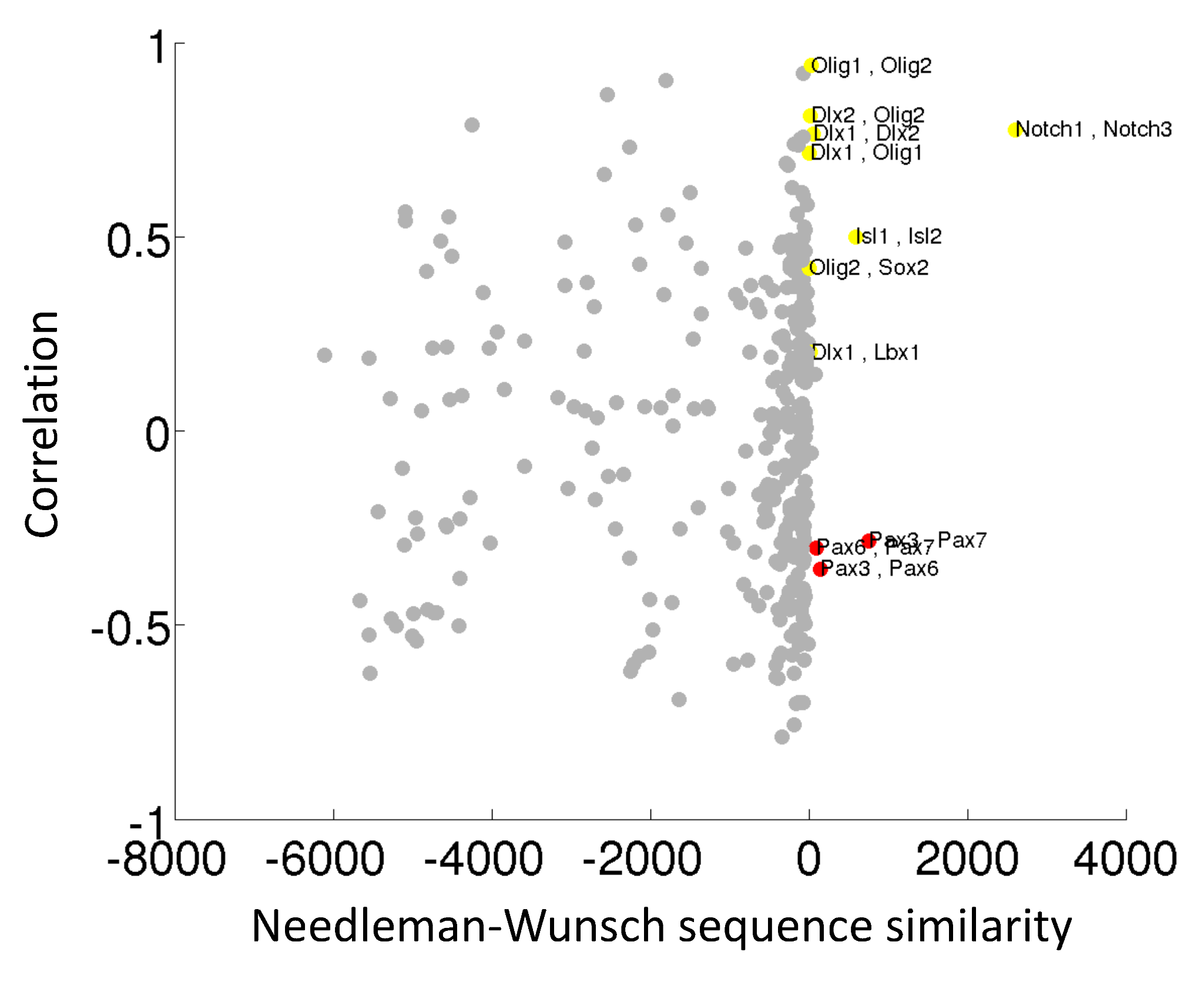

Supplement: Figure S2 — Sequence similarity vs. spatial correlation of gene pairs belonging to the GO category ‘neuron fate commitment’. Pairs of genes with sequence similarity >0 and spatial correlation >0.2 are marked in yellow. Pairs of genes with sequence similarity >0 and spatial correlation <−0.2 are marked in red. (TIF) [file pcbi.1003185.s002.tif]

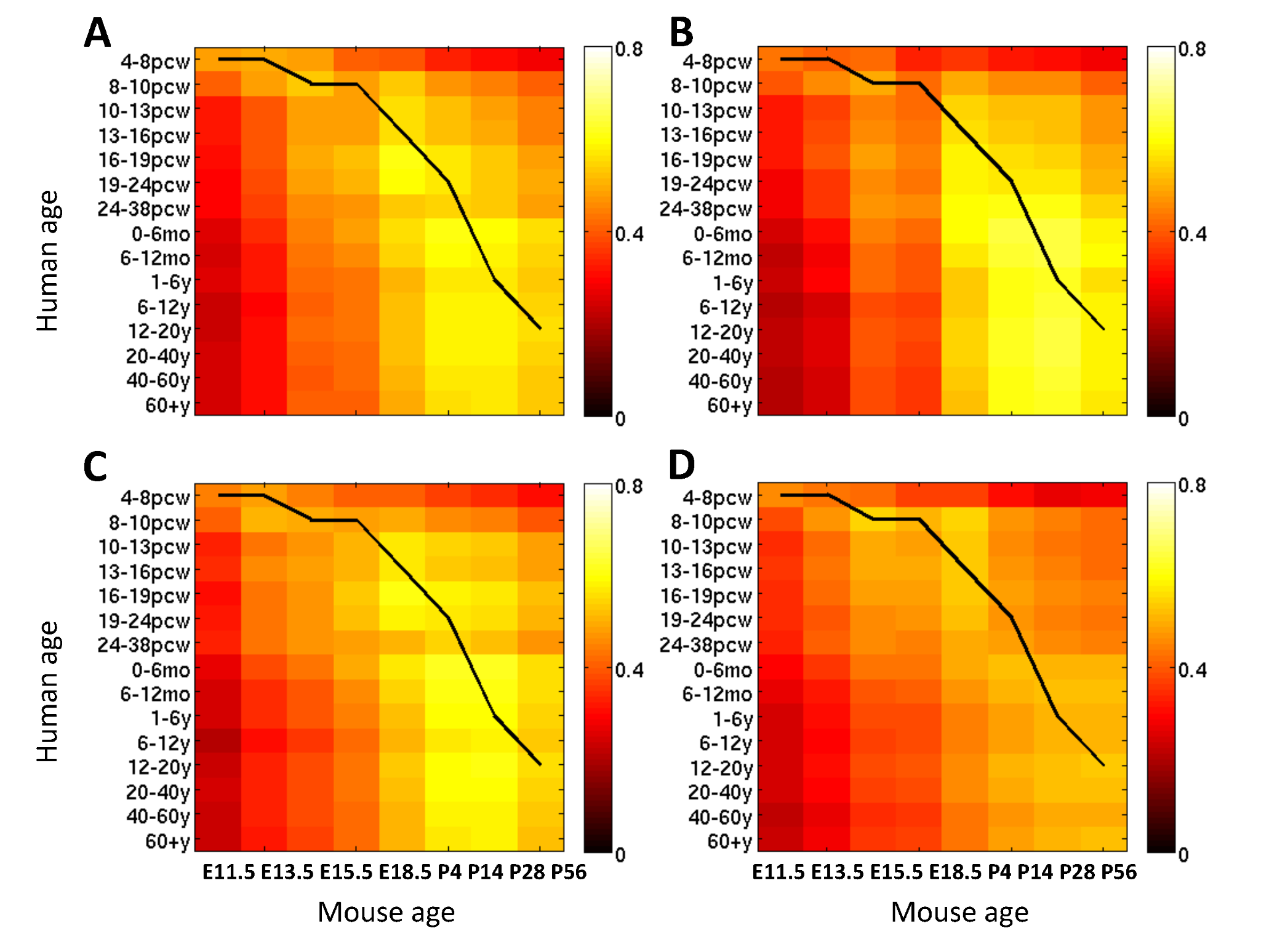

Supplement: Figure S3 — Cross-correlation between mouse and human expression profiles over development. Coherence between expression profiles for orthologous genes was measured using Spearman correlation, for every pair of time points in mouse and human. (A) Thalamus (B) Cortex (C) Striatum and (D) Cerebellum. The black line depicts the mapping between neurodevelopmental timelines of the two species proposed by [27]. (TIF) [file pcbi.1003185.s003.tif]
